# Resveratrol, curcumin, paclitaxel and miRNAs mediated regulation of PI3K/Akt/mTOR pathway: go four better to treat bladder cancer

**DOI:** 10.1186/s12935-020-01660-7

**Published:** 2020-11-23

**Authors:** Khushbukhat Khan, Cristina Quispe, Zeeshan Javed, Muhammad Javed Iqbal, Haleema Sadia, Shahid Raza, Asma Irshad, Bahare Salehi, Željko Reiner, Javad Sharifi-Rad

**Affiliations:** 1 Atta-Ur-Rahman School of Applied Biosciences (ASAB), National University of Sciences and Technology (NUST), Sector H-12, Islamabad, 44000 Pakistan; 2 Facultad de Ciencias de La Salud, Universidad Arturo Prat, Avda. Arturo Prat 2120, 1110939 Iquique, Chile; 3 Lahore Garrison University, Main Campus, Sector C, Phase VI, DHA Lahore Pakistan, Lahore, Pakistan; 4 Department of Biotechnology, Faculty of Sciences, University of Sialkot, Punjab, Pakistan; 5 Department of Biotechnology, BUITMS, Quetta, Pakistan; 6 Department of Life Sciences, University of Management Sciences, Lahore, Pakistan; 7 Medical Ethics and Law Research Center, Shahid Beheshti University of Medical Sciences, Tehran, Iran; 8 Department of Internal Medicine, School of Medicine, University Hospital Centre Zagreb, University of Zagreb, Zagreb, Croatia; 9 Phytochemistry Research Center, Shahid Beheshti University of Medical Sciences, Tehran, Iran

**Keywords:** mTOR, miRNAs, Biomarkers, Natural therapeutic, Drug resistance

## Abstract

Bladder cancer (BC) is a leading cause of death among urothelial malignancies that more commonly affect male population. Poor prognosis and resistance to chemotherapy are the two most important characteristics of this disease. PI3K/Akt/mTOR signaling pathway has been considered pivotal in the regulation of proliferation, migration, invasiveness, and metastasis. Deregulation of PI3K/Akt/mTOR signaling has been found in 40% of bladder cancers. Several microRNAs (miRNAs) have been reported to interact with the PI3K/Akt/mTOR signaling pathway with a different possible role in proliferation and apoptosis in bladder cancer. Thus, miRNAs can be used as potential biomarkers for BC. Natural compounds have been in the spotlight for the past decade due to their effective anti-proliferative capabilities. However, little is known of its possible effects in bladder cancer. The aim of this review is to discuss the interplay between PI3K/Akt/mTOR, miRNAs, and natural compounds and emphasize the importance of miRNAs as biomarkers and resveratrol, curcumin and paclitaxel as a possible therapeutic approach against bladder cancer.

## Introduction

Bladder cancer (BC) is among the nine most prevalent types of cancer globally [1]. The male population is mostly affected by this severe disease as BC is among the seventh most common cancers in males. BC accounts for 165,000 deaths annually, and approximately 430,000 cases are reported each year worldwide [2, 3]. In US alone bladder cancer has been responsible for more than 17,240 deaths in the year 2018 and rate of incidence is increasing suggesting that more and more men are suffering from this disease due to occupational exposure to carcinogens and smoking. Men in the United States are nearly four times more likely to be diagnosed with BC than women and approximately 1 in 27 men in the United States will develop BC in their lifetime. However, in some regions, mortality associated with BC is more frequent in females [2]. The incidence of BC is comparatively less in Asian countries but high mortality rate of BC is reported in Western Asia [4].

BC is a multifaceted disease [5]. The clinical manifestations associated with BC are underpinned by the complex molecular landscape. Cumulative effect of genomic rearrangements, over-expression of oncogenes, loss of tumor suppressor genes and pro-survival signaling pathway integration aggravate this complex anomaly. BC is categorized into non-muscle invasive or muscle invasive carcinoma. Majority of bladder cancers are urothelial carcinomas. Nearly 75% patients are suffering from the non-muscle invasive urothelial carcinoma while 25% cases show the symptoms of muscle invasive metastatic urothelial carcinoma [6]. Non-muscle invasive BC is usually low-grade tumor while muscle invasive tumors are high grade tumors. Morphologically, bladder tumors can be divided into papillary, solid, and mixed types. The papillary type is predominant, especially in non-muscle-invasive bladder cancer [6].

Next generation sequencing in combination with in silico approaches have provided in-depth analysis of cancer genome. This has increased our understanding of genomic alterations occurring inside a tumor cell. These approaches have also outlined the drivers behind the deregulated oncogenic pathways and altered biological mechanism responsible for cancer progression. In addition to this the development of potentially more precise treatment for cancer has become more convenient [7, 8]. Despite these advancements finding an effective treatment for BC is still under developed. Resistance to drugs and disease relapse are responsible for high number of deaths in BC [9].

Additionally, deregulated signaling cascades play a crucial role in the progression of different cancers [10]. Cellular abrasions in the phosphoinositide 3-kinase/AKT/mammalian target of rapamyacin (PI3K/AKT/mTOR) signaling pathway have been considered as a promoting factor in the progression of various cancers [11]. The PI3K/AKT/mTOR pathway has been explored extensively to develop therapeutic strategies for different types of cancers [12, 13]. Several mutations in the PI3K/AKT/mTOR pathway cause irregular growth of cancer cells [14].

PI3K and Akt being upstream factors of mTOR might be suitable therapeutic targets in numerous cancers [15, 16]. Development of effective kinase inhibitors that target signaling cascades at different levels has been in focus of scientists for a number of years. However, since PI3K/Akt signaling pathway plays a crucial role in regulating many important cellular events such as cell proliferation, energy homeostasis and survival; adverse effects usually occur due to the toxicity of these inhibitors to healthy cells [17, 18]. In such a situation, the downstream effectors of the PI3K/Akt pathway such as mTOR, can be a target for the treatment of various cancers. Pathway dysregulation affiliated with PI3K/Akt/mTOR has been reported to be detrimental in more than 50% of all human cancers [19]. Up-regulation of mTOR signaling cascade is responsible for tumorigenesis [20]. Mutations of negative regulators of the mTOR such as the Phosphatase and tensin homolog (PTEN), Tuberous Sclerosis protein (TSC1/2) and mTORC1 complex result in an increased severity of disease and decreased responsiveness to drugs. Based on these data it can be concluded that mTOR can be a potential target for the development of therapeutic strategies for BC.

miRNAs are small molecules (< 20–> 200 bp) that orchestrate development, differentiation, apoptosis, metastasis and cellular homeostasis [21]. Advancements in high throughput technology, along with microarray and RNA-seq are providing a better insight of the complex role of miRNAs in various cellular pathologies including cancer [22]. Several studies have helped to delineate the involvement of miRNA in regulation of the mTOR signaling pathway. The interplay between miRNAs and mTOR plays a crucial role in mTOR mediated cell proliferation and inhibition. A recent study showed the interplay between *miR-99-5p* and mTOR in benzyl isothiocyanate treated BC cells. There was a reciprocal relationship between miR-99-5p and mTOR expression. Microarray based analysis revealed that there were more than 79 miRNAs dysregulated in benzyl isothiocyanate treated BC cells. Among these dysregulated miRNAs, *miR-99-5p* was up-regulated. *miR-99-5p* expression resulted in the decreased expression of the insulin growth factor 1 receptor (IGF1R) and fibroblast growth factor receptor 3 (FGFR3). This study found also a decreased expression of the mTOR confirmed by the qPCR and western blot suggesting that mTOR was directly regulated by the *miR-99-5p*. These findings indicated that *miR-99-5p* has a role in benzyl isothiocyanate mediated inhibition of cellular growth in the BC cells andmiR-99-5p expression helped in cessation of mTOR mediated proliferation in BC [23].

Natural compounds have been extensively used for the treatment of various diseases [24–26]. The anti-cancer efficacy of natural substances has long history. Resveratrol, curcumin and paclitaxel are bioactive molecules with well documented anti-cancer properties. Their interactions with different genes have established them as a modulator of various cancers [27–29]. These compounds help in the regulation of the oxidative stress. Regulation of oxidative stress is an interesting aspect of natural compound mediated anti-cancer activity [27, 30]. Nevertheless, not much has been found about the role of these phytochemicals in the regulation of mTOR mediated signaling. Therefore, this review aims to explain the significant role of mTOR signaling in BC and the interaction between miRNAs and mTOR signaling in BC. The objectives of this review are also to highlight the therapeutic potential of resveratrol, curcumin and paclitaxel and their impact on mTOR signaling in BC.

## mTOR signaling pathway

**Fig. 1:**
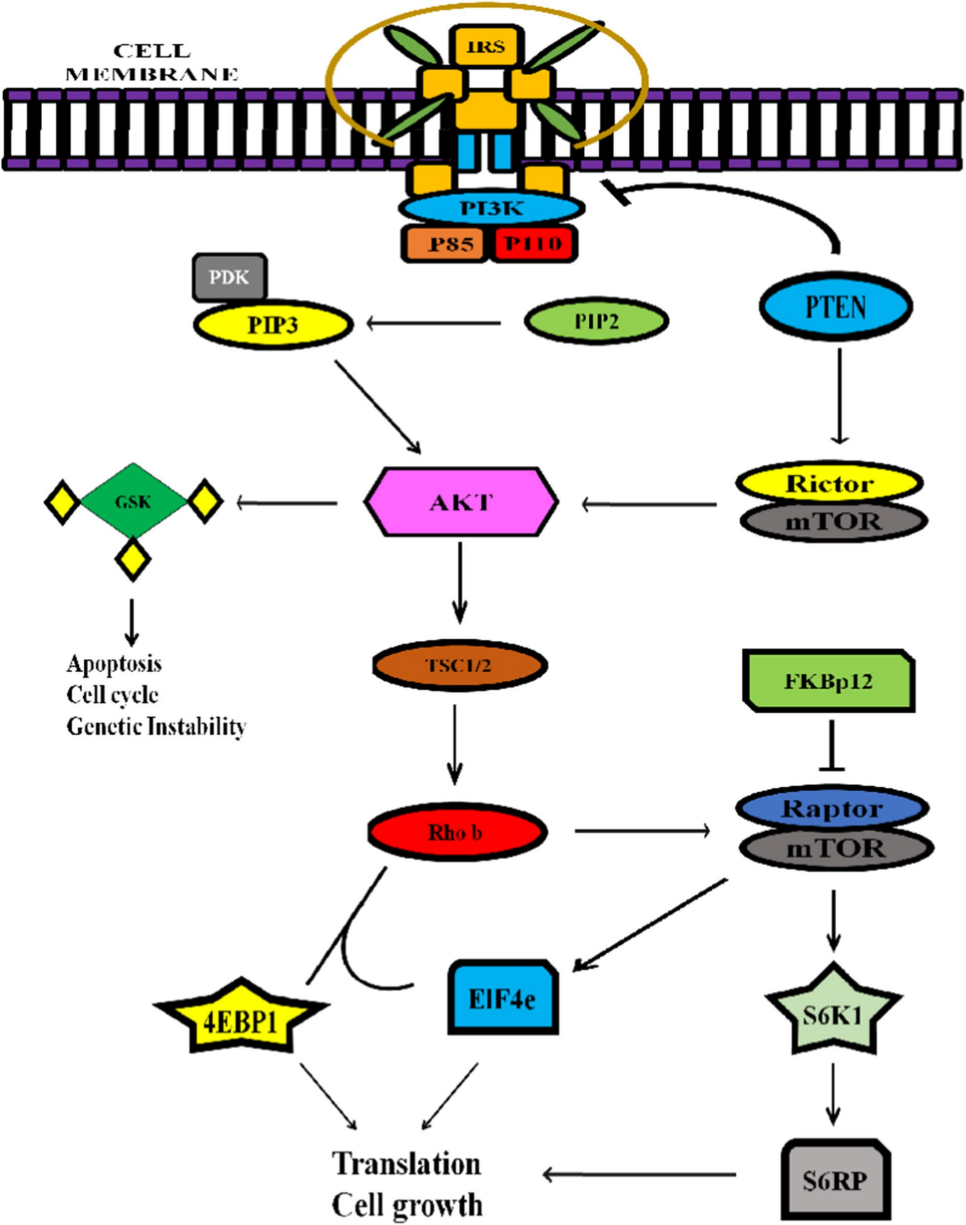
Schematic representation of mTOR signaling cascade. Classical mTOR signaling cascade is triggered by the binding of ligand to insulin receptor that causes the activation of PI3K and finally initiation of signal transduction from mTORC1 and mTORC2 complexes that allows translation of targeted genes and cytoskeleton rearrangements

**Fig. 2:**
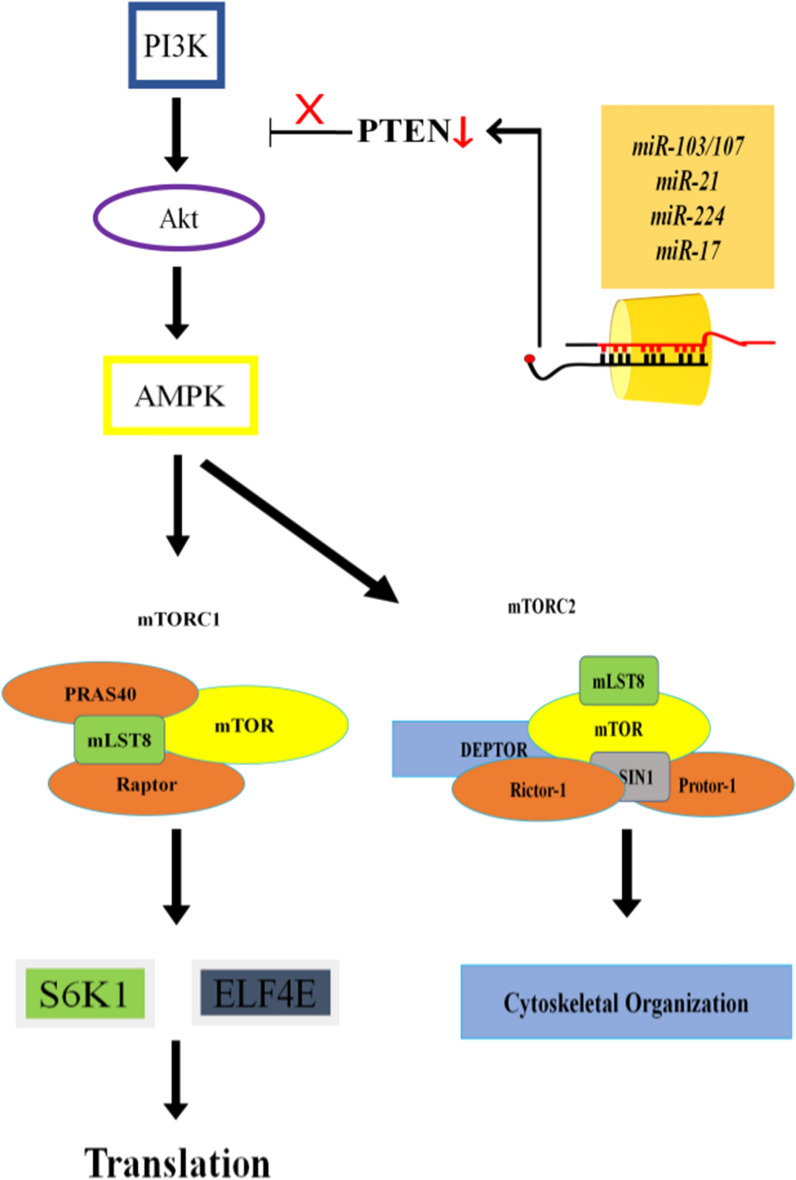
miRNAsis involved in up-regulation of the bladder cancer. Majority of miRNAs involved in proliferation of bladder cancer directly target PTEN, a negative regulator of mTOR pathway. PTEN regulation via miRNAs action allows continuous transduction of Akt/mTOR signaling which causes an abnormal cellular growth and tumor formation. Red arrow in the diagram presents down-regulation while red-cross presents inhibition of signal transduction

The (Mammalian Target of Rapamycin) mTOR protein is an essential protein kinase which seems to regulate many cellular processes including protein synthesis, cell growth, and differentiation. mTOR does this under the influence of growth factors, nutrients and stress [31]. There are two complexes—mTOR complex 1 (mTORC1) and mTOR complex 2 (mTORC2) that constitute the mTOR [32]. The mTOR signaling cascade is presented in Fig. 1. Although the expression of these two complexes is strictly maintained in healthy cells,the deregulation of these complexes is a marker for several metabolic diseases and cancers [33]. Normally, mTOR signaling cascade is triggered by the binding of specific growth factor receptors such as the insulin growth factor (IGF), vascular endothelial growth factor (VEGF), platelet derived epidermal growth factor (PDGF) and epidermal growth factor (EGF). The kinases such as the PI3Ks (Phophoiositide 3 Kinase) have certain regulatory subunits such as the p85 and catalytic subunits p110 that facilitate the catalytic phosphorylation of the (Phosphatidylinositolbiphosphate) PIP2 to PIP3 causing an activation of the signaling cascade [34]. This receptor–ligand interaction promotes the recruitment of the PI3K near receptor site which in turn converts the PIP2 to PIP3 and provides an attachment site for phosphoinositide-dependent kinase 1 (PDK1) [35]. PDK1 attachment to PIP3 promotes the activation of the AKT via phosphorylation. AKT then promotes the activation of several downstream effectors that are involved in the activation of different cellular cascades including mTORC1 causing cellular proliferation. On the other hand, mTORC2 orchestrates activation of AGC family of kinases including AKT, (Serine/threonine protein Kinase 1) SGK1 and (Protein Kinase C) PKC. The targeting of these kinases by various anti-apoptotic drugs inhibits tumor growth and proliferation [36] (Fig. 2).

Metabolism is strictly regulated by the mTOR activation. Any alteration in the signaling cascade causes metabolic changes in glucose, lipids and fatty acids metabolism. Tumor cells are always exposed to stress and therefore they require an additional amount of energy for their survival. Aberrant mTOR signaling provides the cancer cells with abundant amount of energy [12]. Dysregulated glucose metabolism results in increased synthesis of glucose transporter proteins and glycolytic enzyme activation. Glucose transporter 1 (GLUT-1) has been associated with deregulation of them TOR signaling cascade. In most types of cancer GLUT-1 is over-expressed suggesting t that GLUT-1 over-expression under the influence of interrupted mTOR signaling cascade results in the activation of oncogenes such as the c-MYC and HIF-1α [37]. GLUT-1 is member of GLUT family that plays a regulatory role in the homeostasis of glucose by monitoring its intake into the cell’s cytoplasm. Glycogen synthase Kinase 3 (GSK-3) negatively regulates the expression of the GLUT-1 gene. Tuberous sclerosis complex (TSC) and mTOR positively regulate the expression of GLUT-1 [38]. mTOR-mediated up-regulation of glucose promotes the process of glycolysis via activation of the hexokinase-2 (HK2) that triggers the phosphorylation of c-MYC and Hypoxia inducing factor 1 alpha(HIF-1α) [39]. The metabolism of fatty acids is also deregulated by the inhibition of mTOR signaling cascade. mTOR signaling cascade induces the synthesis of fatty acids by activation of the sterol regulatory element binding protein 1c (SREBP) [40].

## Modulators of mTOR signaling in bladder cancer

mTOR signaling pathway is necessary for cellular functions for which it needs a huge amount of nutrients and energy. For mTOR pathway to work, AKT is first recruited to plasma membrane where it is phosphorylated at T308 and S473 sites. This in turn results in the activation of AKT. mTORC2 primarily acts as AKT S473 kinase while mTORC1, after being activated by AKT, is essential for cell growth and metabolism modulation. mTOR erroneous signaling is suppressed by its two negative regulators: TSC1 and TSC2 [41]. Mutations in any of these substances cause hyper-activation of mTOR pathway. It has been reported that loss of heterozygosity (LOH) of chromosome 9 occurs in > 50% of BC which also has to do with TSC1. Alteration in TSC1 causes mTORC1 signaling up-regulation, leading to tumor formation [42, 43].

Mitogen Activated Protein Kinase-4 (MAPK-4) in different types of cancers is identified as an activating agent for AKT/mTOR signaling via alternative pathway. MAPK-4 activates AKT by phosphorylating it at T308 and it also activates mTORC2 which then fully activates AKT. Activation of AKT promotes cell proliferation and resistance to PI3K inhibitor treatment. MAPK-4 over-expression in BC is directly associated with shorter patients’ survival. Knocking down of MAPK-4 induces inhibition of tumor growth and re-sensitization to PI3K inhibitor [44].

In aggressive BCa, transmembrane protein- glycoprotein 130 (GP130) is highly expressed. In an in vivo experiment, its high expression is reported to have a direct association with elevated phosphorylation of mAKT and mTOR, implying it as an obvious cause for rapid tumor growth and aggressiveness. Knocking down of GP130 via siRNA containing nano-particles caused a decrease in cell viability, tumor growth and migration in T24 and UM-UC-3 BC cell lines which suggested that gp130 might be a therapeutic target against tumor growth [45].

Sperm-associated antigen 5 (SPAG5) plays a vital role in mitosis regulation. Its high expression has been reported in both BC tissue and BC cell lines by Liu et al. Its high concentration correlated with higher levels of p-AKT and p-mTOR which suggested that it might have a role in enhanced BC size and growth bythe activation of AKT/mTOR signaling pathway. Moreover, aberrant expressions of SPAG-5 are responsible for poor survival of BC patients [46].

mTOR signaling transduction in BC is also amplified by the up-regulated expression of sororin (CDCA5)—a protein essential for sister chromatid separation and cohesion. Its high expression has been correlated with poor survival. Sororin inhibits intrinsic apoptosis and promotes cell proliferation by activating PI3K/AKT/mTOR pathway and also by enhancing expression of cyclinD and CDC2 [47].

In physiological conditions, mTOR helps the cell’s growth and metabolism through mTORC1 signaling. mTORC1, after being activated, induces the accumulation of transcription factor SREBP‐1c in nucleus, which is a pre-requisite step for de novo synthesis of fatty acids and cholesterol. Both fatty acids and cholesterol are necessary for different cellular processes and growth. SREBP-1 cup-regulates fatty acids synthase (FASN) expression. FASN is essential for the catalysis of final steps of fatty acids de novo synthesis and its high expression in BCis directly associated with cancer development, patients’ shorter survival and higher probability of the relapsing of disease [48, 49]. Pyruvate kinase M2 (PKM-2) is recently discovered to be a modulator of SREBP-1c. PKM-2 physically binds with SREBP-1c to activate it. Its over-expression in BC has been reported by Su et al. [50]. It has been demonstrated in in vitro experiment that the down-regulation of PKM-2 causes reduction in SREBP-1c expression via modulation of AKT/mTOR signaling. PKM-2 inhibition blocks the phosphorylation of AKT and mTOR which reduces SREBP-1c expression and ultimately, suppresses FASN transcriptional activation. Moreover, PKM-2 inhibition also inhibits cell growth and promotes cell apoptosis [48].

mTORC2 induces stabilization and maturation of PKCs by phosphorylating them at their turn motifs and hydrophobic motifs [51]. PKCs hyperactivation in numerous types of cancers including BC is associated with cell growth, angiogenesis and metastasis. Patel et al. treated TCCSUP cells with atypical PKC inhibitors and rapamycin and reported attenuated viability of BC cells [52].Their study suggested that mTOR pathway plays a role in PKCs activation and also suggested a possible combined therapeutic approach for BC.

Long non-coding RNAs contribution in BC initiation and progression by modulating mTOR signaling has been also demonstrated. The over-expression of DUXAP10 in BC tissues is associated with increased cancer growth and proliferation. Knocking down of DUXAP10 in T24 and 5637 cells negatively influenced the cell growth. Mechanistically, it inhibited phosphorylation-activation of Akt which ultimately, prevented the phosphorylation of mTOR. DUXAP10 knock-down also up-regulated expression of PTEN which induced cell cycle arrest at G1/G0 phase, inhibited cell proliferation and facilitated apoptosis by increasing expression of pro-apoptotic proteins [53].

## MiRNA-mediated regulation of mTOR in bladder cancer

Several microRNAs are discovered which play important roles in modulating vital processes in cells including cellular growth and apoptosis. These miRNAs maintain the normal balance of cellular growth. Dysregulation in their expression causes uncontrolled cellular growth which then can turn into carcinogenesis. After tumor formation, miRNA also support cancer growth and metastasis [54–56]. Like is the case with other cancers, miRNA contribution in tumorigenesis in bladder has been documented as well. These miRNAs by regulating different pathways, including mTOR signaling pathway, play an important role in BC initiation, progression and metastasis (Table 1) [57, 58]. Figure 2 presents the interplay of different miRNAs in modulating mTOR signaling in BC.

**Table 1:** List of miRNAsand their target genes, along with their expression in bladder cancer

miRNA	Target gene	Expression	BC cell line		References
			Name	Type	
*miR-103*	PTEN	Up-regulated	UMUC2, 5637		[60]
*miR-146a*	AMPKα	Up-regulated	T24, 5637	Muscle invasive	[66]
*miR-21*	PTEN	Up-regulated	T24, 5637	Muscle invasive	[103]
*miR-224*	PTEN	Up-regulated	EJ, T24, 253 J, RT4, TCC-SUP, UMUC, J82, 5637		[68]
*miR-218*	BMI-1	Up-regulated	T24, EJ		[69]
*miR-222*	PPAR2A	Up-regulated	T24, 5637		[70]
*miR-99a-5p*	S6KI	Down-regulated	J82, HT‐1376, 5637, RT4, and T24		[62]
*miR-100*	PTEN	Down-regulated	CaBER, BIU-87, 5637, T24, T4, J82, HT-1376, EJ, TCCSUP, SV4		[65]
*miR-126*	PI3KR2	Down-regulated	BLS		[71]
*miR-125-5p*	Hexokinase2	Down-regulated	T24, RT4, J82, 5637		[73]

**Table 2:** List of natural compounds and their target genes as well as their expression in bladder cancer

Natural compound	Cell line	Target gene	Expression	References
Resveratrol	T24, ECV-304	Bcl-2	Down-regulate	[88, 89]
	639Vs	mTORC1	Down-regulate	[93]
	RT4, 5637, T24	Akt, mTOR, SRC	Down-regulate	[102, 103]
	5637	Akt	Down-regulate	[105]
		PTEN	Up-regulate	
Curcumin	T24	P53	Down-regulate	[118]
	T24, J82, TCCSUP	Akt, SRC	Down-regulate	[119]
	EJ	PI3K	Suppress activation	[126]
Paclitaxel	T24	Akt	Down-regulate	[134]

MiR-103 and miR-107 are two tumor-promoting microRNAs in BC which have similar seed sequence and can only be distinguished by one base [59]. Elevated expression of miR-103/107 in BC cells facilitates the rapid cell proliferation by activating PI3K/AKT/mTOR cascade. Yu et al. inhibited miR-103/107 by transfecting antagomiR-103/107 in UMUC2 and 5637 human bladder cell lines. They have showed using western blot analysis that inhibition of miR-103/107 significantly reduced the AKT phosphorylation-activation. As a result, the activation of mTOR was also prevented which was dependent on Akt activation. miR-103/107 duo has its cell proliferating effect by blocking the expression of PI3K/AKT signaling pathway negative regulator—PTEN [60]. According to the results of Han et al. [61], reduced PTEN level in BC cells is associated with tumor aggressiveness.

Among all microRNAs, miR-99a-5p plays an essential role in modulation of mTOR/AKT signaling. It directly targets mTOR where it facilitates in the suppression of cancerous cell proliferation by binding to 3′UTR of mTOR mRNA at position 295–301 of nucleotide [62, 63]. It has been demonstrated that reduced expression of miR-99a-5p was n directly associated with BCand was responsible for poor survival rate of patients [62]. The miR‐99a‐5p/mTOR axis induces growth inhibitory effect on BC cells by negative regulation of S6K1 phosphorylation. S6K1, a key translation regulator, is major target of mTOR and has a pivotal role in cancerous cells’ growth and proliferation [64]. Another group of scientists induced expression of miR-99a-5p in 5637 and T24 BC cell lines by transfecting miR-99a expression-vector and demonstrated that miR-99a-5p re-expression also prevented mTORC1 and mTORC2 signaling transduction. Since mTOR is a core component of both complexes, down-regulation of mTOR causes a modulation of mTORC1 and mTORC2. These studies indicate the importance of miR-99a-5p in controlling tumor growth in BC [63]. miR-100 is another miRNA which suppresses tumor growth by directly targeting mTOR. By using the quantitative real-time PCR, Xu et al. have found that miR-100 is under-expressed in BC tissue and in BC cell lines. They further evaluated tumor-suppressor role of miR-100 by it’s ectopic restoration in BC cell lines and reported that its re-expression caused cell-cycle arrest and inhibited proliferation and motility of cancer cells [65].

MicroRNAs contribution in chemo-resistance and in increasing number of cancer cells is well documented. Zhuang et al. reported about the direct association of exosomal miR-146a and enhanced cancer initiating properties and chemo-resistance in BC where it has an important role by post-transcriptionally repressing AMPKα and promoting mTOR signaling. They further reported that miR-146a is highly expressed in BC cells and is secreted by cancer-associated fibroblasts at muscle invasive stage [66]. The findings of this study highlight the potential significance of miR-146a as a prognostic marker for predicting the probability of cancer recurrence and cancer stage. MiR-21 is another microRNA which has its tumorigenic influence on transitional cell carcinoma of BC by down-regulating PTEN and inducing chemo-resistance to doxorubicin. miR-21 is highly over-expressed in cancer cells and due to consequent negative-regulation of PTEN, increased rate of phosphorylation of AKT and up-regulation of anti-apoptotic protein BCL-2, cell proliferation is increased [67]. PTEN is direct target for another microRNA-miR-224 or miR-17. The increased level of these miRNAs in BC cells correlates with lower expression of circular RNA circ-ITCH which is important for increased cell proliferation. circ-ITCH over-expression sequesters miR-17 or miR-224 and enhanced PTEN expression in BC cell lines. In vitro over-expression of circ-ITCH promoted cell apoptosis while in vivo over-expression induced suppression of xenografted tumor growth [68]. PTEN is also indirectly modulated by miR-218 in BC cells. miR-218 targets BMI-1 mRNA in non-cancerous cells but its lower expression in BC cells is the reason for BMI-1 over-expression. PTEN, being the down-stream target of BMI-1, is suppressed which allows cancerous cells to replicate. Over-expression of miR-218 inhibits BMI-1 and restores PTEN expression which consequently inhibits tumor cell proliferation and migration [69].

MiR-222 is responsible for resistance to cisplatinin BC. It is highly expressed in BC cells and decreases apoptotic response of cisplatin by activating AKT/mTOR signaling pathway and also facilitates the induction of cellular proliferation. miR-222 activates mTOR signaling by inhibiting PPP2R2A at post-transcriptional phase and its lower expression then reciprocally activates PI3K/Akt/mTOR pathway [70].

Some microRNAs have tumor-suppressing role in cancers and their expression is down-regulated in most types of cancers including BC. miR-126 is one these microRNAs whose decreased expression in BC is associated with increased cell proliferation, migration and invasiveness [71]. Multiple targets of miR-126 have been discovered until now and their modulation by miR-126 has negative effects on BC cell viability. For instance, down-regulation of ADAM-9 mRNA causes inhibition of cell invasiveness [72] and by targeting PIK3R2 gene it modulates PI3K/Akt pathway and has a negative impacton cell’s proliferation [71]. MiR-125b-5p is another tumor suppressive miRNA. Its lower expression directly correlates with shorter survival time and metastases. Its ectopic expression in T24 and J82 cells reduced cell viability, decreased metastatic potential and promoted cell death. Luciferase reporter assay, immunoblotting and qRT-PCR suggested thathexokinase2 might be a direct target of miR125b-5p. Hexokinase2 is down-stream target of PI3K/Akt pathway. Western blot analysis showed that enhanced miR125-5p expression also decreases the phosphorylation activation of Akt and PI3K [73]. miR125-5p can directly or through PI2K/Akt pathway inhibit hexokinase 2 which causes tumor growth and metastasis suppression.

Zhai and Xu demonstrated that the lower levels of miR-126 in T24 cells have direct correlation with high expression of lncRNA ATB. They further reported that ATB binds complementarily with miR-126 and prevents its binding with target mRNA. KRAS is another target of miR-126 and its down-regulation by lncRNA ATB induces elevation in KRAS expression which activates AKT/mTOR signaling pathway by promoting phosphorylation-activation of PI3K, AKT and mTOR [56]. Re-expression of miR-126 by the transfection of recombinant-lentivirus vector in BLS cells facilitated apoptosis and suppressed tumor proliferation and migration [71]. This suggests the possible therapeutic importance of miR-126 in BC.

Study in 2014 reported attenuated expression of four papillary BC (pBC)-specific miRNAs. The expression of miRNAs, miR-200c, miR-205, miR-145 and miR-125 was significantly lower in high garde pBC. Further, negative correlation between miR-205/miR-200c and ZEB1/2 was demonstrated which are the downstream effector targeted for Akt [74].

mTOR pathway role in autophagy is well-documented. miRNAs also regulates mTOR induced autophagy progress. In BC this process is inhibited by miR-21 which inhibits PTEN, caspase-3, beclin-1 and E-cadherin causing cell proliferation, invasion and migration as well as inhibition of apoptosis [75]. Wang et al. reported the autophagy promoting role of sodium butyrate (NaB) in BC. NaB applied to BC cells promoted up-regulation of miR-139-5p that induced activation of autophagy via AMPK/mTOR pathway and caused overproduction of ROS. As a consequence the viability of cancer cells was reduced and the process of caspase-dependent apoptosis was activated. MiR-139-5p targets Bmi-1 which induces reversion of invasion and migration [76]. Hydroxycamptothecinals initiated apoptosis via promoting AMPK/mTOR pathway dependent apoptosis [77], but the exact involvement of miRNAs in this process is not yet known.

## Natural compounds as possible therapeutic solution

Natural compounds have been used for the treatment of different anomalies for thousand years [78]. Plants produce bioactive secondary metabolites that exclusively protect them from predators. These bioactive compounds have been used for the treatment of cancer as well [79]. Few derivatives of plant-based phytochemicals have gained prominence in the field of cancer biology because of their limited cytotoxic effects and high specificity. Among these bioactive compounds resveratrol, curcumin, quercetin, paclitexal, genistein, Epigallocatechin-3-gallate (EGCG) and several others have been investigated extensively for their antitumor properties [80]. Phytochemicals have limited side effects which make them safe for the administration as proven in animal models and cell lines. These compounds interact with different molecules and signaling cascades that alter molecular landscape to tumor cells. Recently obtained data from cutting edge research have shown that deregulated signaling are critical hallmarks of BC. Accumulating scientific evidence has helped to delineate the interplay between miRNAs and natural compounds in hampering cellular growth in bladder cancer. Here we summarize few of these compounds and their interaction with miRNAs in regulation of BC and can be implemented as possible therapeutic option (Table 2).

## Resveratrol

**Fig. 3:**
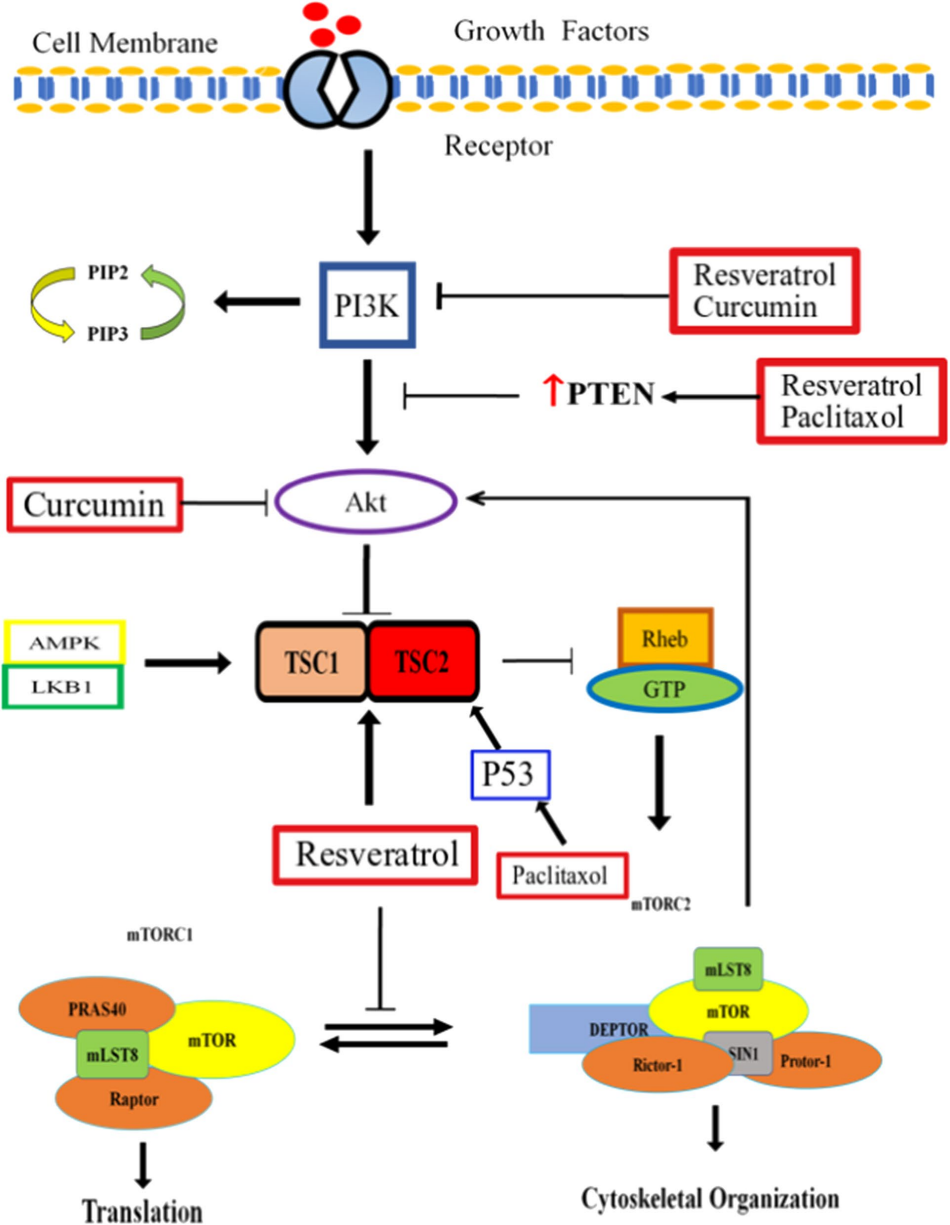
Resveratrol, curcumin and paclitaxol affects the mTOR pathway at several stages from inhibition to PI3K to suppression of Akt signaling to prevent the mTORC1 and mTORC2 complexes. Paclitaxol also stabilizes P53 which promotes complex formation of TSC1 and TSC2. TSC1/2 complex then represses mTOR signaling. These compounds mediated inhibition of mTOR complexes results in the apoptosis and stop of bladder cancer growth

Resveratrol (chemically 3,5,4′-trihydroxystilbene) is a naturally-occurring edible poly-phenolic anti-oxidant which could mostly be found in red wine, barriers, peanuts and grapes [81]. Several studies have demonstrated its chemo-preventive effects concerning tumor initiation, growth and progression [82–84]. It specifically inhibits the growth of cancer cells, leaving healthy cells unaffected [85, 86]. It triggers apoptosis by impairing oxidative phosphorylation and inducing over-production of ROS [87]. In BC cells, it induces dose-dependent inhibitory effect on Akt phosphorylation at S473. Akt inactivation negatively influences the phosphorylation of Bcl-2 agonist of cell death (BAD) at Ser112 and Ser136. Resveratrol also induces Akt phosphorylation reduction and increases phosphorylation of p38 MAPK. Since MAPK is negative-regulator of cyclin D1 and CDK, its activation results in cell cycle arrest at G1 phase. Resveratrol promotes apoptosis in BC cells by down-regulating Bcl-2 and Bcl-xL expression and by up-regulating Bax expression. It also induces cleavage of caspase-3 and PARP in a dose-dependent manner [88]. Stocco et al*.* demonstrated the dose-dependent inhibitory effect of resveratrol on ECV304 cells. They reported that low concentrations of resveratrol had negligible inhibitory effect on cancer cells. On the contrary, it provided protection to cells against oxidative stress. Nevertheless, high concentrations of resveratrol modulated Bcl-2 expression and enhanced cell damage and apoptosis rate [89]. The efficacy of resveratrol cytotoxic effect is dose- as well as time-dependent. Its high concentrations (~ 150-200 μM) induce growth arrest after 2 h of resveratrol exposure and apoptosis after 72 h of exposure in human TCC EJ cells [90]. Its time-dependent cytotoxic effect is also recorded in T24 and BTT39 cell lines too. Its cytotoxic effects include caspase 3- and 9- cleavage activation, disruption of mitochondrial membrane, cytochrome c release in cytosol and increased production of ROS [91]. Yang et al. reported that different cell lines of BC differ in their sensitivity to resveratrol. They evaluated the sensitivity of T24 and EJ cells for resveratrol and have found that despite producing similar metabolic profile, T24 were more sensitive to resveratrol. Their finding provided significant insight regarding resveratrol therapeutic potential specifically when individual differences are taken into account [92]. Schematic representation of resveratrol effects on mTOR signaling which causes changed cellular growth and tumor formation is presented in Fig. 3.

Alayev et al. evaluated mTOR signaling regulation based anti-cancer properties in BC cell line by exposing it to resveratrol and rapamycin co-treatment. They reported that treatment with resveratrol alone or in combination with rapamycin efficiently inhibited the cell growth by inducing growth stop by targeting Akt activation and preventing mTORC1 signaling cascade phosphorylation-activation. Their findings suggested that resveratrol might be a potential option for BC treatment [93].

Resveratrol therapeutic potential also includes its ability to treat BC resistant to numerous drugs either by re-sensitizing cancer cells to certain drug or by inhibiting cell growth by adopting alternative apoptosis route [94–97]. It re-sensitized cancer cells to doxorubicin in a dose-dependent manner [96, 98, 99]. It has a pro-apoptotic effect on taxol resistant BC cell lines by inhibiting cell growth beyond S-phase [100]. Among several drugs investigated, Wang et al. reported that resveratrol was most efficient in inducing apoptosis in adriamycin (ADM) resistant pumc91 BC cells. They also reported that resveratrol caused significant reduction in expression of MRP-1, GST, BCL-2 and LRP and an increase in expression of Topo-II [101].

In a recent study, the effect of resveratrol in BC cells varying in TP53 gene status was analyzed. Resveratrol caused a reduction in cell proliferation promoted cellular damage in all cell lines. Downregulation of Akt, mTOR and SRC expression, regulation of DNMT1 expression and an increased rate of cell death was reported in wild type P53 cells. Cell cycle arrest at S phase occurred in mutated TP53 cells while HOXB3/RASSF1A pathway modulation along with PCNA levels reduction in nucleus occurred in the highest‐grade cells [102].

**Fig. 4:**
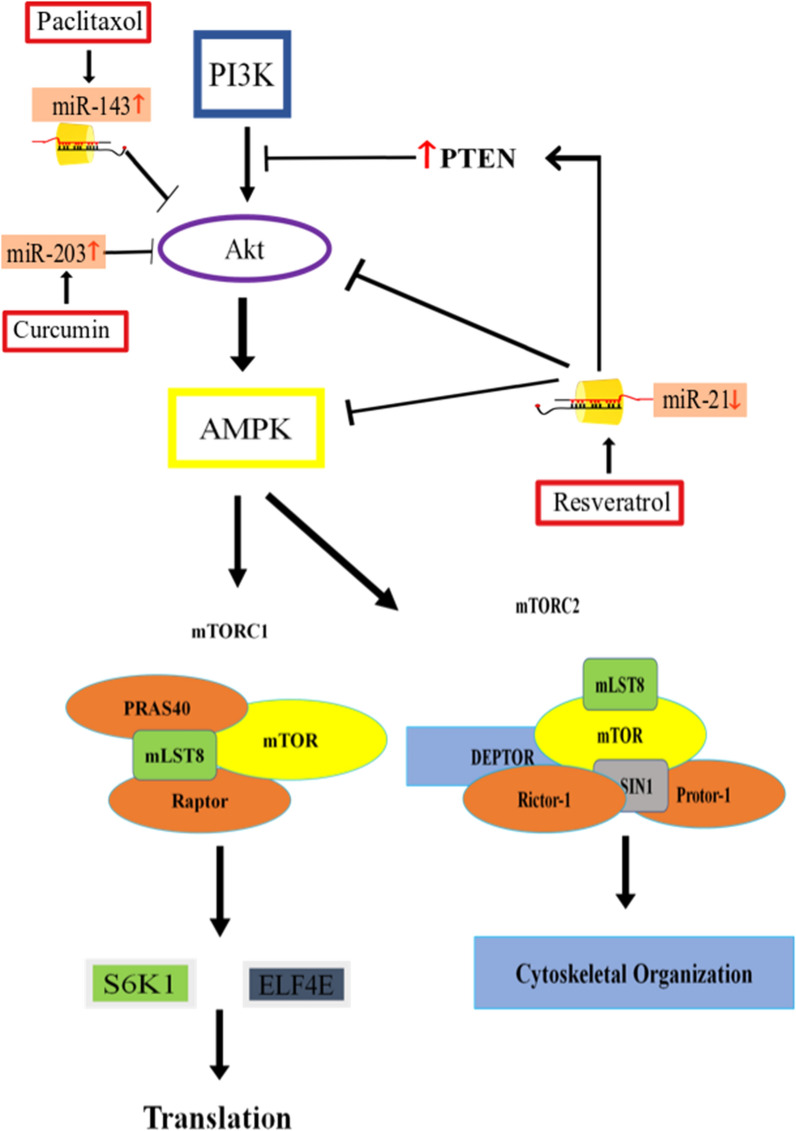
MiRNAs under the influence of resveratrol, curcumin and paclitaxel efficiently inhibit the downstream signaling cascades that in turn promote the apoptosis and stop cell growth. These compounds mediated signaling up-regulates PTEN expression, inhibits Akt activation and also regulates activation of mTOR signaling-downstream effector molecules. Red up-arrow in diagram presents up-regulation while red down-arrow presents down-regulation

Resveratrol’s modulating Akt/mTOR signaling by regulating microRNA expression was investigated by Zhou et al. in BC5637 and T24 cells. According to their findings, resveratrol down-regulates the expression of miR-21 [103]—a microRNA that induces resistance to doxorubicin treatment, prevents apoptosis and facilitates uncontrolled cell proliferation [67, 103, 104]. Resveratrol-induced down-regulation of miR-21 also caused a reduction in Akt phosphorylation which reduced cell proliferation (Fig. 4). It also down-regulated Bcl-2 protein expression and enhanced caspase-3 activation which promoted apoptosis [103]. Resveratrol also facilitates apoptosis by negatively regulating mRNA and protein levels of Akt and enhancing PTEN mRNA and protein levels [105]. Similarly, this compound also induces apoptosis in gastric cancer cells by stopping Akt-PI3K signaling via up-regulating PTEN expression [106]. However, in hepatocellular carcinoma resveratrol does not induce apoptosis by modulating PTEN expression [107]. These findings suggest possible participation of another molecule which resveratrol utilizes to modulate PTEN levels. In prostate cancer, microRNAs miR-17, miR-20a and miR-106b are targeted by resveratrol by which it up-regulates PTEN levels [108, 109].

Despite its efficient anti-cancer response, scientists face the limitation of its poor bioavailability, instability and hydrophobicity which lowers its efficiency. Current investigations are focused on overcoming these limitations by using its micro- or macro encapsulations. This technique enhances resveratrol’s stability and the release of its dose is also controlled [110]. Resveratrol conjugation to gold nanoparticles is also studied with respect to its stability and bioavailability [111]. Lipid nano carries are also investigated as transport tool for resveratrol [112]. All these methods have enhanced its stability and improved its bioavailability but further experimental endeavours are essential to first unravel these technique’s benefits and second to develop efficient delivery system for these encapsulations.

## Curcumin

Curcumin is a polyphenol obtained from the Curcuma longa. Curcumin has broad range pharmacological properties [113]. Phenolic extracts of curcumin has been reported to have anti-cancer, anti-inflammatory, anti-oxidant and anti-microbial properties [114]. Curcumin exerts its anti-proliferative role via modulation of the gene expression of different oncogenes which inhibit cellular growth and trigger apoptosis. Curcumin regulates the expression of several signaling cascades such as the Nuclear factor Kappa B, Akt, MAPK and others. In addition to curcumin, miRNAs also modulate the expression of key signaling pathways. For instance, in human pancreatic cells, curcumin modulates the expression of miRNA-22 and miRNA-199a*. Curcumin interacts with SP1 transcription factor and estrogen receptor and thus promotes apoptosis. miRNA-22 is up-regulated by the curcumin while miR-199a* is down-regulated resulting in the inhibition of growth in pancreatic cancer [115]. Administration of curcumin, piperine and taurine down regulated the expression of the miR-21 and interleukin-10 in hepatocellular carcinoma patients. The anti-tumor effect of curcumin results in the regulation of the miR-21. miR-21 modulation in turn facilitates cell cycle regulation and apoptosis. miR-21 overexpression down-regulates the expression PTEN and PDCD4 protein in hepatocellular carcinoma [116]. In vivo and in vitro studies have further confirmed the anti-proliferative role of curcumin in various cancers. Curcumin has also been involved in the treatment of melanoma but further studies are required to elaborate the clear mechanism behind its pro-apoptotic effect and clinical efficacy [117]. In bladder cancer, curcumin has been reported to regulate the expression of miR-1246 which enhances radio-sensitization of tumor cells. miR-1246 under the influence of the curcumin targets P53 [118]. Curcumin has been reported to regulate epigenetic modification in breast cancer via modulation of the expression of miR-203. In bladder cancer cell lines, it was observed that curcumin up-regulated the expression of miR-203 and in turn decreased the expression of AKT2 and SRC. Down-regulation of the Akt2 and Src promoted apoptosis in bladder cancer cells [119]. These findings indicate the importance of curcumin as possible therapeutic solution for bladder cancer. Curcumin downregulates the expression of miR-7641 and thus increases the expression of p16 which in turn reduces invasiveness and increases apoptosis in bladder cancer cells [120]. Nearly 40% bladder cancers have abrogated activation of the PI3K/AKT/mTOR pathway [121, 122]. Poor survival rate is major risk factor associated with mTOR signaling aberrations in bladder cancer [123, 124]. It has been reported in the rat bladder carcinogenesis model that curcumin impedes the PI3K/Akt/mTOR signaling pathway. In addition to this the investigators postulated that curcumin directly inhibits the expression of insulin-like growth factor (IGF2) and prevents the phosphorylation of the insulin receptor substrate 1 (IRS-1) a prerequisite for PI3K signaling initiation thus blocks cellular growth and proliferation [125]. Another recent study has delineated the link between curcumin administration and mechanistic down-regulation of proto-oncogene c-MYC. The investigators suggested that curcumin in EJ bladder cancer cells prevents the activation of PI3K thus suppresses the downstream activation of mTOR and constitutive activation of proto-oncogenes such as the c-MYC [126].

The stability and bioavailability of curcumin is a major concern. These shortcomings lower its chemopreventive advantages. Like resveratrol, nano technology has assisted in overcoming its limitations but extensive research is still needed to develop better delivery systems for these nano carries and to lower cytotoxicity of nano particles [127, 128].

## Paclitaxel

Paclitaxel is obtained from the bark of the western yew plant and possess anti-leukemic and anti-tumor properties [129]. Several preclinical studies have demonstrated the anti-proliferative role of paclitaxel in different cancers such as the ovarian, breast, lung and sarcomas such as the kaposi’s sarcoma. Paclitaxel has been employed as an adjuvant for treating tumors related to gastroesophageal, cervical, prostate, head and neck and endometrial tissues [130]. Despites its involvement as a potent chemotherapeutic agent there is one major concern regarding the administration of paclitaxel (paclitaxel resistance). This has greatly hampered its application as chemotherapeutic agent. However, miRNAs can reduce the paclitaxel resistance. It has come to limelight less lately that miR-200c can overcome paclitaxel resistance via modulation of the cathepsin L (CTSL)- mediated epithelial-mesenchymal transition in A549 cells via feedback loop system [131]. Also, paclitaxel resistance was reversed by the overexpression of miR-107 in A549/Taxol cells. Overexpression of the miR-107 significantly reduced the expression of Bcl-w in A549/Taxol cells [132]. However, interaction of paclitaxel with miRNAs in bladder cancer and regulation of mTOR still requires substantial studies. Paclitaxel in combination with the geridonin inhibited cell growth and promote mitochondrial apoptosis in gastric cancer cell lines. Paclitaxel and gerdonin when administrated synergistically resulted in the up-regulation of PTEN that recruited P53 and suppressed growth of the gastric cancer cells. Activated PTEN prevented phosphorylation of the Akt and murine double minute 2 (MDM2) protein thus inhibited growth [133]. Owing to these findings’ paclitaxel can be used as a therapeutic approach in synergy with geridonim for gastric as well as bladder cancer. In bladder cancer, pacliaxel up-regulates the expression of miR-143 which post-transcriptionally inhibits the expression of Akt [134].

The anti-tumor properties of these compounds in bladder cancer are well-studied but data regarding its influence on mTOR signaling modulating miRNAs require further investigations to better understand its mechanism of action. Improved understanding can facilitate their possible use in BC treatment.

## Clinical trials on resveratrol, curcumin or paclitaxel in cancer

Experiment evidences on different cancer cell lines and animal models have extensively proved the chemotherapeutic and chemopreventive significance of resveratrol, curcumin and paclitaxel. However, the efficacy of these compounds should be evaluated in human patients. Few papers in current decade have been published which highlighted the clinical use of these natural compounds in different cancers. According to these studies, the therapeutic intervention of all three natural compounds strongly depended on cancer stage and type and drug dosage and treatment period.

Curcumin clinical trials in different cancers are extensively done [135]. Clinical trials regarding curcumin treatment in bladder cancer demonstrated that 8000 mg/day of its dose for 3 months confer no side effects and has also attenuated bladder cancer lesions [136]. Likewise, its 3.6 g/day dose is pharmacologically beneficial in reducing the concentration of oxidative DNA adducts in colorectal cancer [137]. In another study, the administration of 180 mg of curcumin effectively attenuated the expression of glutathione S-transferase (GST) activity in colorectal cancer [138]. Major concern in adoption of curcumin at clinical level is its poor bioavailability. Despite this limitation, several clinical trials in numerous cancers have shown its anticancer activity [135, 139].

Paclitaxel was evaluated for single agent activity in phase 2 oncology group trials, Eastern Cooperative, A dose of 20 mg/m^2^ was infused in 2 voluntary patients. Paclitaxel activity was reported in transitional BC cells [140]. In another study, paclitaxel was co-treated with gemcitabine in 102 advanced BC patients. In Phase 3 of treatment trial, it was reported that prolonged treatment of these drugs induced toxicity in patients but this strategy can be used as second line of treatment against metastatic BC [141]. Randomized phase III trial was conducted to analyse paclitaxel/gemcitabine/cisplatin in invasive BC. The outcomes showed that the adjuvant enhanced overall survival. But, the study was prematurely shut down due to which its actual outcomes remain inconclusive [142]. Recently, albumin-bound formulation of paclitaxel was also investigated in 199 patients in phase 2 randomized clinical trial. Paclitaxel formulation had similar therapeutic influence as paclitaxel, however, paclitaxel formulation led to more toxicity then paclitaxel [143].

The clinical trials of resveratrol are on initial phases [144]. Pharmacokinetics and pharmacodynamics of resveratrol in 6 hepatic metastasized colon cancer individuals was investigated. In this phase 1 double-blind study, 5 g dose was given for 2-weeks. The miniature quantity of compound along with high expression of caspase 3 was detectable in malignant liver tissue [145]. Another clinical evaluation was performed on 20 colon cancer patients in which 0.5 to 1 g of resveratrol dose was reported safe and effective in curbing tumor proliferation by 5% [146]. In 2015, Kjær and colleagues evaluated two doses, 150 mg and 1000 mg, of resveratrol on 66 middle-aged patients of prostate cancer in placebo controlled-randomized clinical study. They found resveratrol-treatment of no significance for benign prostate cancer [147]. In same year, phase 1 clinical study on therapeutic influence of MPX (Muscadine Naturals. Inc., Clemmons, NC), a skin extract of *Vitis rotundifolia*, on 14 patients of biochemically recurrent prostate cancer (BRPC) was published. The extract consisted of resveratrol, ellagic acid and quercetin. According to the study, 4000 mg dose of MPX accompanied no adverse effect, was effective in 50% of the patients and delayed recurrence of BRPC by lengthening the doubling time of PSA [148]. Early trial in high-risk breast cancer women showed that resveratrol promoted demethylation of tumor-suppressive RASSF-1α gene. Contrarily to these studies, resveratrol treatment in patients of multiple myeloma led to renal toxicity and eventually renal failure [149]. This toxicity is multiple myeloma specific; yet, its treatment outcomes in human trials did not proven its much clinical significance in cancers. In colon and breast cancer, more human trials might prove successful in evaluating its full beneficial potential. Despite showing chemotherapeutic influence in bladder cancer cell lines and xerographs, its potential in human bladder cancer patients is not yet investigated.

Evidences from clinical trials indicate that these three natural compounds modulated NF-κB signaling, STATs associated signaling and glutamine pathway [135, 150]. But none of the clinical trials investigated their regulatory influence on mTOR pathway. Therefore, current information available [125–127, 131–135] on underlying mechanism of action of these compounds is incomplete. Trials in bladder cancer are never done to analyze the therapeutic potential of resveratrol. One preliminary study regarding curcumin role in bladder carcinoma human subjects is not enough to delineate its capacity. Contrarily to curcumin and resveratrol, promising data exist for paclitaxel anticancer influence in bladder cancer. But none of the study highlighted the underlying mechanism of action of this compound. So, further experimental evaluation is necessary to understand the paclitaxel mechanism of anticancer action, to further validate curcumin potential and targeted pathway and to investigate the role of resveratrol in BC human patients.

## Conclusion

Deregulation of the controlled cell signaling pathways trigger tumor progression and metastasis. Genomic and proteomic approaches can help to understand the underlying mechanisms involved in development and progression of BC. These approaches have helped in identification of novel possible anticancer treatment approaches such as miRNAs and natural compounds. Reduced apoptosis is a characteristic of aggressive cancer cells and phytochemicals re-balances pro- and anti-apoptotic proteins to improve the efficacy of TRAIL-based therapeutics. Recent studies have started to give an insighton the role of miRNAs as regulators of cell division, metastasis and growth arrest. Therefore, miRNAs might be used as a biomarker target for various types of cancer. Additionally, natural compounds such as resveratrol, curcumin and paclitaxel seem to play a crucial role in the modulation of the multiple proteins of oncogenic pathways. The anti-tumor effects of resveratrol, curcumin and paclitaxel have been reported based upon the inhibition of the TGF/SMAD, NFκ-B, PI3K/Akt/mTOR, NOTCH and JAK-STAT pathways. Resveratrol inhibits the phosphorylation of the SMADs that in turn inhibit cell growth and metastasis. Resveratrol-mediated inhibition of mTOR has also recently been addressed. mTORC1 and mTORC2 are the two downstream effectors of the PI3K/Akt/mTOR pathway that has been modulated by the resveratrol. The consequence is beneficial effects on curbing cellular growth and invasiveness in BC. Resveratrol efficiently upregulates different tumor suppressor miRNAs in different types of cancer, and its suppressive effects on oncogenic miRNAs have also been well documented. Moreover, resveratrol in combination with the miR-99-5p prevents cellular growth in vitro. miR-99-5p, miR-100 and miR-126 are the three major miRNAs that regulate the expression of the PI3K, S6K and PTEN thus inhibiting cell growth and metastasis in BC. Curcumin has been extensively employed in the cancer treatment at least in cell lines. This has led to speculation that curcumin can be a potential therapeutic target for various cancers in near future. However, circumstantial research efforts are needed to put curcumin in to the therapeutic avenues. Curcumin has been reported to regulate the expression various miRNAs both at epigenetics and translational levels. Owing to these characteristics’ curcumin based nano formulations can be employed for specific drug delivery to the target tissues. Curcumin based nanoformualtions are under clinical trials and can be implemented for various cancers. However, there are several hurdles such as the dosage, bioavailability, toxicity, optimal indication and potential side effects which require attention. Resolving these issues can put curcumin on the list of possible therapeutic options for BC. Paclitaxel has been efficiently used in chemotherapy over the years but the major stumbling block regarding the administration of paclitaxel is drug resistance. New cutting-edge research has shed light on the approaches which can reduce paclitaxel mediated drug resistance and increase its bioavailability. However, this requires more in vivo and in vitro evidences. The interactions between paclitaxel, PI3K/Akt/mTOR and miRNAs in BC provide significant insight on the pathology of disease which also open new venues for treating this cancer. Yet, more extensive studies on further elucidating these interactions are required, only than a possible and more effective therapeutic can be built.

Studies till date have demonstrated the individual influence of these natural compounds on BC. In future studies demonstrating cumulative influence of resveratrol, curcumin, paclitaxel or their derivatives could provide another possible therapeutic use of these compounds in BC. Keeping on mind the multitargeted approach of resveratrol, curcumin, paclitaxel and miRNAs and their robust anticancer effects that regulate many cellular processes, these substances could be considered an important molecular and pharmacological effective armament against BC.

## Data Availability

Yes.

## References

[1] Antoni S, Ferlay J, Soerjomataram I, Znaor A, Jemal A, Bray F (2017). Bladder cancer incidence and mortality: a global overview and recent trends. Eur Urol.

[2] Siegel RL, Miller KD, Jemal A (2018). Cancer statistics. CA Cancer J Clin.

[3] Kogevinas M. Bladder cancer. In: Occupational cancers. Berlin: Springer; 2020, p. 487–506.

[4] Wong MC, Fung FD, Leung C, Cheung WW, Goggins WB, Ng C (2018). The global epidemiology of bladder cancer: a joinpoint regression analysis of its incidence and mortality trends and projection. Sci Rep.

[5] Scicinski J, Kashfi K. Cancer and beyond: discovery and development of NO-releasing therapeutics. In: Therapeutic application of nitric oxide in cancer and inflammatory disorders. Amsterdam: Elsevier; 2019. p. 123–158.

[6] Kamat AM, Hahn NM, Efstathiou JA, Lerner SP, Malmström P-U, Choi W, Guo CC, Lotan Y, Kassouf W (2016). Bladder cancer. Lancet.

[7] Pietzak EJ, Bagrodia A, Cha EK, Drill EN, Iyer G, Isharwal S, Ostrovnaya I, Baez P, Li Q, Berger MF (2017). Next-generation sequencing of nonmuscle invasive bladder cancer reveals potential biomarkers and rational therapeutic targets. Eur Urol.

[8] Osterhout R, Kamal A, Spigelman S (2019). Identification of novel targets in non-muscle invasive bladder cancer: a systems biology approach. Am Soc Clin Oncol..

[9] David D, Abufaraj M, Susani M, Ristl R, Foerster B, Kimura S, Mari A, Soria F, Briganti A, Karakiewicz PI (2018). Accurate prediction of progression to muscle-invasive disease in patients with pT1G3 bladder cancer: a clinical decision-making tool. Urol Oncol.

[10] Signore M, Ricci-Vitiani L, De Maria R (2013). Targeting apoptosis pathways in cancer stem cells. Cancer Lett.

[11] Youssef M, Cuddihy A, Darido C (2017). Long-lived epidermal cancer-initiating cells. Int J Mol Sci.

[12] Mossmann D, Park S, Hall MN (2018). mTOR signalling and cellular metabolism are mutual determinants in cancer. Nat Rev Cancer.

[13] Tan FH, Bai Y, Saintigny P, Darido C (2019). mTOR signalling in head and neck cancer: heads up. Cells.

[14] Arafeh R, Samuels Y (2019). PIK3CA in cancer: the past 30 years. Semin Cancer Biol..

[15] Ciuffreda L, Di Sanza C, Incani UC, Milella M (2010). The mTOR pathway: a new target in cancer therapy. Curr Cancer Drug Targets.

[16] Jiang W, Ji M (2019). Receptor tyrosine kinases in PI3K signaling: the therapeutic targets in cancer. Semin Cancer Biol..

[17] Hay N, Sonenberg N (2004). Upstream and downstream of mTOR. Genes Dev.

[18] Guertin DA, Sabatini DM (2005). An expanding role for mTOR in cancer. Trends Mol Med.

[19] Vivanco I, Sawyers CL (2002). The phosphatidylinositol 3-kinase—AKT pathway in human cancer. Nat Rev Cancer.

[20] Zha X, Hu Z, He S, Wang F, Shen H, Zhang H (2011). TSC1/TSC2 inactivation inhibits AKT through mTORC1-dependent up-regulation of STAT3-PTEN cascade. Cancer Lett.

[21] Javed Z, Iqbal MZ, Latif MU, Yaqub HMF, Qadri QR (2015). Potent implications of miRNA in cancer biology—a brief review. Adv Life Sci.

[22] Farooqi AA, Fayyaz S, Shatynska-Mytsyk I, Javed Z, Jabeen S, Yaylim I, Gasparri ML, Panici PB (2016). Is miR-34a a well-equipped swordsman to conquer temple of molecular oncology?. Chem Biol Drug Des.

[23] Lin JF, Tsai TF, Lin YC, Chen HE, Chou KY, Hwang TI (2019). Benzyl isothiocyanate suppresses IGF1R, FGFR3 and mTOR expression by upregulation of miR-99a-5p in human bladder cancer cells. Int J Oncol.

[24] Salehi B, Selamoglu Z, Sener B, Kilic M, Kumar Jugran A, de Tommasi N, Sinisgalli C, Milella L, Rajkovic J, Morais-Braga FB (2019). Berberis plants—drifting from farm to food applications, phytotherapy, and phytopharmacology. Foods.

[25] Salehi B, Sener B, Kilic M, Sharifi-Rad J, Naz R, Yousaf Z, Mudau FN, Fokou PVT, Ezzat SM, El Bishbishy MH (2019). Dioscorea plants: a genus rich in vital nutra-pharmaceuticals—a review. Iran J Pharm Res..

[26] Sharifi-Rad J, Melgar-Lalanne G, Hernández-Álvarez AJ, Taheri Y, Shaheen S, Kregiel D, Antolak H, Pawlikowska E, Brdar-Jokanović M, Rajkovic J (2020). Malva species: insights on its chemical composition towards pharmacological applications. Phytotherapy Res.

[27] Farooqi A, Khalid S, Ahmad A (2018). Regulation of cell signaling pathways and miRNAs by resveratrol in different cancers. Int J Mol Sci.

[28] Rutz J, Janicova A, Woidacki K, Chun FKH, Blaheta RA, Relja B (2020). Curcumin—a viable agent for better bladder cancer treatment. Int J Mol Sci.

[29] Hernández-Prat A, Rodriguez-Vida A, Juanpere-Rodero N, Arpi O, Menéndez S, Soria-Jiménez L, Martínez A, Iarchouk N, Rojo F, Albanell J (2019). Novel oral mTORC1/2 inhibitor TAK-228 has synergistic antitumor effects when combined with paclitaxel or PI3Kα inhibitor TAK-117 in preclinical bladder cancer models. Mol Cancer Res.

[30] Wu Y, Liu F (2013). Targeting mTOR: evaluating the therapeutic potential of resveratrol for cancer treatment. Anti-Cancer Agents Med Chem (formerly Current Medicinal Chemistry-Anti-Cancer Agents)..

[31] Fonseca BD, Graber TE, Hoang H-D, González A, Soukas AA, Hernández G, Alain T, Swift SL, Weisman R, Meyer C. Evolution of TOR and translation control. In: Evolution of the protein synthesis machinery and its regulation. Berlin: Springer; 2016. p 327–411.

[32] Efeyan A, Sabatini DM (2010). mTOR and cancer: many loops in one pathway. Curr Opin Cell Biol.

[33] Pearce LR, Huang X, Boudeau J, Pawłowski R, Wullschleger S, Deak M, Ibrahim AF, Gourlay R, Magnuson MA, Alessi DR (2007). Identification of protor as a novel rictor-binding component of mTOR complex-2. Biochem J.

[34] Engelman JA, Luo J, Cantley LC (2006). The evolution of phosphatidylinositol 3-kinases as regulators of growth and metabolism. Nat Rev Genet.

[35] Laplante M, Sabatini DM (2009). mTOR signaling at a glance. J Cell Sci.

[36] Mamane Y, Petroulakis E, LeBacquer O, Sonenberg N (2006). mTOR, translation initiation and cancer. Oncogene.

[37] Buller CL, Loberg RD, Fan M-H, Zhu Q, Park JL, Vesely E, Inoki K, Guan K-L, Brosius FC (2008). A GSK-3/TSC2/mTOR pathway regulates glucose uptake and GLUT1 glucose transporter expression. Am J Physiol Cell Physiol.

[38] Szablewski L (2013). Expression of glucose transporters in cancers. Biochimica et Biophysica Acta (BBA)—Rev Cancer.

[39] Kleszcz R, Paluszczak J, Krajka-Kuźniak V, Baer-Dubowska W (2018). The inhibition of c-MYC transcription factor modulates the expression of glycolytic and glutaminolytic enzymes in FaDu hypopharyngeal carcinoma cells. Adv Clin Exp Med.

[40] Ricoult SJ, Yecies JL, Ben-Sahra I, Manning BD (2016). Oncogenic PI3K and K-Ras stimulate de novo lipid synthesis through mTORC1 and SREBP. Oncogene.

[41] Kim J, Guan K-L (2019). mTOR as a central hub of nutrient signalling and cell growth. Nat Cell Biol.

[42] Hansel DE, Platt E, Orloff M, Harwalker J, Sethu S, Hicks JL, De Marzo A, Steinle RE, Hsi ED, Theodorescu D (2010). Mammalian target of rapamycin (mTOR) regulates cellular proliferation and tumor growth in urothelial carcinoma. Am J Pathol.

[43] Knowles MA, Platt FM, Ross RL, Hurst CD (2009). Phosphatidylinositol 3-kinase (PI3K) pathway activation in bladder cancer. Cancer Metastasis Rev.

[44] Wang W, Shen T, Dong B, Creighton CJ, Meng Y, Zhou W, Shi Q, Zhou H, Zhang Y, Moore DD (2019). MAPK4 overexpression promotes tumor progression via noncanonical activation of AKT/mTOR signaling. J Clin Investig.

[45] Martin DT, Shen H, Steinbach-Rankins JM, Zhu X, Johnson KK, Syed J, Saltzman WM, Weiss RM (2019). Glycoprotein-130 expression is associated with aggressive bladder cancer and is a potential therapeutic target. Mol Cancer Ther.

[46] Liu J, Zeng Q, Cao P, Xie D, Yang F, He L, Dai Y, Li J, Liu X, Zeng H (2018). SPAG5 promotes proliferation and suppresses apoptosis in bladder urothelial carcinoma by upregulating Wnt3 via activating the AKT/mTOR pathway and predicts poorer survival. Oncogene.

[47] Fu G, Xu Z, Chen X, Pan H, Wang Y, Jin B (2020). CDCA5 functions as a tumor promoter in bladder cancer by dysregulating mitochondria-mediated apoptosis, cell cycle regulation and PI3k/AKT/mTOR pathway activation. J Cancer.

[48] Tao T, Su Q, Xu S, Deng J, Zhou S, Zhuang Y, Huang Y, He C, He S, Peng M (2019). Down-regulation of PKM2 decreases FASN expression in bladder cancer cells through AKT/mTOR/SREBP-1c axis. J Cell Physiol.

[49] Jiang B, Li E-H, Lu Y-Y, Jiang Q, Cui D, Jing Y-F, Xia S-J (2012). Inhibition of fatty-acid synthase suppresses P-AKT and induces apoptosis in bladder cancer. Urology.

[50] Su Q, Tao T, Tang L, Deng J, Darko KO, Zhou S, Peng M, He S, Zeng Q, Chen AF (2018). Down-regulation of PKM 2 enhances anticancer efficiency of THP on bladder cancer. J Cell Mol Med.

[51] Ikenoue T, Inoki K, Yang Q, Zhou X, Guan KL (2008). Essential function of TORC2 in PKC and Akt turn motif phosphorylation, maturation and signalling. EMBO J.

[52] Patel R, Islam S, Bommareddy RR, Smalley T, Acevedo-Duncan M (2020). Simultaneous inhibition of atypical protein kinase-C and mTOR impedes bladder cancer cell progression. Int J Oncol.

[53] Lv X-Y, Ma L, Chen J-F, Yu R, Li Y, Yan ZJ, Cheng Y, Ma Q (2018). Knockdown of DUXAP10 inhibits proliferation and promotes apoptosis in bladder cancer cells via PI3K/Akt/mTOR signaling pathway. Int J Oncol.

[54] Zhang L, Zhang X, Wang X, He M, Qiao S. MicroRNA-224 promotes tumorigenesis through downregulation of caspase-9 in triple-negative breast cancer. Dis Mark. 2019. 10.1155/2019/7378967 PMC6388334 30886656

[55] Saleh A, Cheng H, Martin SE, Si H, Ormanaglu P, Carlson SG, Clavijo PE, Yang X, Das R, Cornelius S (2019). Integrated genomic and functional microRNA analysis identifies miR-30-5p as a tumor suppressor and potential therapeutic nanomedicine in head and neck cancer. Clin Cancer Res..

[56] Zhai X, Xu W (2018). Long noncoding RNA ATB promotes proliferation, migration, and invasion in bladder cancer by suppressing microRNA-126. Oncol Res Featur Preclin Clin Cancer Ther.

[57] Chen L, Long Y, Han Z, Yuan Z, Liu W, Yang F, Li T, Shu L, Zhong Y (2019). MicroRNA-101 inhibits cell migration and invasion in bladder cancer via targeting FZD4. Exp Ther Med.

[58] Jiang H, Bu Q, Zeng M, Xia D, Wu A (2019). MicroRNA-93 promotes bladder cancer proliferation and invasion by targeting PEDF. Urol Oncol Semin Orig Investig..

[59] Scheffer A-R, Holdenrieder S, Kristiansen G, von Ruecker A, Müller SC, Ellinger J (2014). Circulating microRNAs in serum: novel biomarkers for patients with bladder cancer?. World J Urol.

[60] Yu Q, Liu P, Li Z, Zhang C, Chen S, Li Z, Zhang G, Li J (2018). MiR-103/107 induces tumorigenicity in bladder cancer cell by suppressing PTEN. Eur Rev Med Pharmacol Sci.

[61] Han KS, Jeong IG, Joung JY, Yang SO, Chung J, Seo HK, Kwon KS, Park WS, Lee KH (2008). Clinical value of PTEN in patients with superficial bladder cancer. Urol Int.

[62] Liu Y, Li B, Yang X, Zhang C (2018). MiR-99a-5p inhibits bladder cancer cell proliferation by directly targeting mammalian target of rapamycin and predicts patient survival. J Cell Biochem.

[63] Tsai T-F, Lin J-F, Chou K-Y, Lin Y-C, Chen H-E (2018). Hwang TI-S: miR-99a-5p acts as tumor suppressor via targeting to mTOR and enhances RAD001-induced apoptosis in human urinary bladder urothelial carcinoma cells. OncoTargets Therapy.

[64] Kwon JK, Kim SJ, Kim JH, Lee KM, Chang IH (2014). Dual inhibition by S6K1 and Elf4E is essential for controlling cellular growth and invasion in bladder cancer. Urol Oncol Semin Orig Investig..

[65] Xu C, Zeng Q, Xu W, Jiao L, Chen Y, Zhang Z, Wu C, Jin T, Pan A, Wei R (2013). miRNA-100 inhibits human bladder urothelial carcinogenesis by directly targeting mTOR. Mol Cancer Ther.

[66] Zhuang J, Shen L, Yan J, Guo H (2017). Cancer-associated fibroblasts secreted exosomal miR-146a promotes bladder cancer progression. Eur Urol Suppl.

[67] Tao J, Lu Q, Wu D, Li P, Xu B, Qing W, Wang M, Zhang Z, Zhang W (2011). microRNA-21 modulates cell proliferation and sensitivity to doxorubicin in bladder cancer cells. Oncol Rep.

[68] Yang C, Yuan W, Yang X, Li P, Wang J, Han J, Tao J, Li P, Yang H, Lv Q (2018). Circular RNA circ-ITCH inhibits bladder cancer progression by sponging miR-17/miR-224 and regulating p21, PTEN expression. Mol Cancer.

[69] Cheng Y, Yang X, Deng X, Zhang X, Li P, Tao J, Lu Q (2015). MicroRNA-218 inhibits bladder cancer cell proliferation, migration, and invasion by targeting BMI-1. Tumor Biol.

[70] Zeng LP, Hu ZM, Li K, Xia K (2016). miR-222 attenuates cisplatin-induced cell death by targeting the PPP 2R2A/Akt/mTOR Axis in bladder cancer cells. J Cell Mol Med.

[71] Xiao J, Lin H-Y, Zhu Y-Y, Zhu Y-P, Chen L-W (2016). MiR-126 regulates proliferation and invasion in the bladder cancer BLS cell line by targeting the PIK3R2-mediated PI3K/Akt signaling pathway. OncoTargets Therapy.

[72] Jia A, Castillo-Martin M, Bonal D, Sánchez-Carbayo M, Silva J, Cordon-Cardo C (2014). MicroRNA-126 inhibits invasion in bladder cancer via regulation of ADAM9. Br J Cancer.

[73] Liu S, Chen Q, Wang Y (2020). MiR-125b-5p suppresses the bladder cancer progression via targeting HK2 and suppressing PI3K/AKT pathway. Hum Cell.

[74] Lee H, Jun S-Y, Lee Y-S, Lee HJ, Lee WS, Park CS (2014). Expression of miRNAs and ZEB1 and ZEB2 correlates with histopathological grade in papillary urothelial tumors of the urinary bladder. Virchows Arch.

[75] Zhang HH, Huang ZX, Zhong SQ, Fei KL, Cao YH (2020). miR-21 inhibits autophagy and promotes malignant development in the bladder cancer T24 cell line. Int J Oncol.

[76] Wang F, Wu H, Fan M, Yu R, Zhang Y, Liu J, Zhou X, Cai Y, Huang S, Hu Z (2020). Sodium butyrate inhibits migration and induces AMPK-mTOR pathway-dependent autophagy and ROS-mediated apoptosis via the miR-139-5p/Bmi-1 axis in human bladder cancer cells. FASEB J.

[77] Wang F, Cao M, Fan M, Wu H, Huang W, Zhang Y, Hu Z, Jin X (2020). AMPK-mTOR-ULK1 axis activation-dependent autophagy promotes hydroxycamptothecin-induced apoptosis in human bladder cancer cells. J Cell Physiol.

[78] Kratz JM, Grienke U, Scheel O, Mann SA, Rollinger JM (2017). Natural products modulating the hERG channel: heartaches and hope. Nat Product Rep.

[79] Kusari S, Pandey SP, Spiteller M (2013). Untapped mutualistic paradigms linking host plant and endophytic fungal production of similar bioactive secondary metabolites. Phytochemistry.

[80] Naujokat C, McKee DL (2020). The “Big Five” phytochemicals targeting cancer stem cells: curcumin, EGCG, sulforaphane, resveratrol, and genistein. Curr Med Chem..

[81] Biesalski HK (2007). Polyphenols and inflammation: basic interactions. Curr Opin Clin Nutr Metab Care.

[82] Li D, Wang G, Jin G, Yao K, Zhao Z, Bie L, Guo Y, Li N, Deng W, Chen X (2019). Resveratrol suppresses colon cancer growth by targeting the AKT/STAT3 signaling pathway. Int J Mol Med.

[83] Yang Z, Xie Q, Chen Z, Ni H, Xia L, Zhao Q, Chen Z, Chen P (2019). Resveratrol suppresses the invasion and migration of human gastric cancer cells via inhibition of MALAT1-mediated epithelial-to-mesenchymal transition. Exp Ther Med.

[84] Kumar A, Levenson AS. Epigenetic mechanisms of resveratrol and its analogs in cancer prevention and treatment. In: Epigenetics of cancer prevention. Elsevier; 2019. p 169–186.

[85] Khan MA, Chen H-C, Wan X-X, Tania M, Xu A-H, Chen F-Z, Zhang D-Z (2013). Regulatory effects of resveratrol on antioxidant enzymes: a mechanism of growth inhibition and apoptosis induction in cancer cells. Mol Cells.

[86] Amiri A, Asemi Z, Shafiee A, Hajighadimi S, Moradizarmehri S, Mirzaei HR, Mirzaei H (2020). Role of resveratrol in modulating microRNAs in human diseases: from cancer to inflammatory disorder. Curr Med Chem.

[87] Rodríguez-Enríquez S, Pacheco-Velázquez SC, Marín-Hernández Á, Gallardo-Pérez JC, Robledo-Cadena DX, Hernández-Reséndiz I, García-García JD, Belmont-Díaz J, López-Marure R, Hernández-Esquivel L (2019). Resveratrol inhibits cancer cell proliferation by impairing oxidative phosphorylation and inducing oxidative stress. Toxicol Appl Pharmacol.

[88] Bai Y, Mao QQ, Qin J, Zheng XY, Wang YB, Yang K, Shen HF, Xie LP (2010). Resveratrol induces apoptosis and cell cycle arrest of human T24 bladder cancer cells in vitro and inhibits tumor growth in vivo. Cancer Sci.

[89] Stocco B, Toledo K, Salvador M, Paulo M, Koyama N, Toloi MRT (2012). Dose-dependent effect of resveratrol on bladder cancer cells: chemoprevention and oxidative stress. Maturitas.

[90] Wu M-L, Li H, Yu L-J, Chen X-Y, Kong Q-Y, Song X, Shu X-H, Liu J (2014). Short-term resveratrol exposure causes in vitro and in vivo growth inhibition and apoptosis of bladder cancer cells. PLoS ONE.

[91] Lin X, Wu G, Huo WQ, Zhang Y, Jin FS (2012). Resveratrol induces apoptosis associated with mitochondrial dysfunction in bladder carcinoma cells. Int J Urol.

[92] Yang Y, Li C, Li H, Wu M, Ren C, Zhen Y, Ma X, Diao Y, Ma X, Deng S (2017). Differential sensitivities of bladder cancer cell lines to resveratol are unrelated to its metabolic profile. Oncotarget.

[93] Alayev A, Salamon RS, Schwartz NS, Berman AY, Wiener SL, Holz MK (2017). Combination of rapamycin and resveratrol for treatment of bladder cancer. J Cell Physiol.

[94] El-Readi MZ, Eid S, Abdelghany AA, Al-Amoudi HS, Efferth T, Wink M (2019). Resveratrol mediated cancer cell apoptosis, and modulation of multidrug resistance proteins and metabolic enzymes. Phytomedicine.

[95] Díaz-Chávez J, Fonseca-Sánchez MA, Arechaga-Ocampo E, Flores-Pérez A, Palacios-Rodríguez Y, Domínguez-Gómez G, Marchat LA, Fuentes-Mera L, Mendoza-Hernández G, Gariglio P (2013). Proteomic profiling reveals that resveratrol inhibits HSP27 expression and sensitizes breast cancer cells to doxorubicin therapy. PLoS ONE.

[96] Jin X, Wei Y, Liu Y, Lu X, Ding F, Wang J, Yang S (2019). Resveratrol promotes sensitization to Doxorubicin by inhibiting epithelial-mesenchymal transition and modulating SIRT1/β-catenin signaling pathway in breast cancer. Cancer Med.

[97] Mohammed S, Harikumar KB. Role of resveratrol in chemosensitization of cancer. In: Role of nutraceuticals in cancer chemosensitization*.* Elsevier; 2018. p 61–76.

[98] Huang F, Wu X-N, Chen J, Wang W-X, Lu ZF (2014). Resveratrol reverses multidrug resistance in human breast cancer doxorubicin-resistant cells. Exp Ther Med.

[99] Guo Y, Zhang H, Xie D, Hu X, Song R, Zhu L (2018). Non-coding RNA NEAT1/miR-214-3p contribute to doxorubicin resistance of urothelial bladder cancer preliminary through the Wnt/β-catenin pathway. Cancer Manag Res.

[100] Mao QQ, Bai Y, Lin YW, Zheng XY, Qin J, Yang K, Xie LP (2010). Resveratrol confers resistance against taxol via induction of cell cycle arrest in human cancer cell lines. Mol Nutr Food Res.

[101] Wang S, Meng Q, Xie Q, Zhang M (2017). Effect and mechanism of resveratrol on drug resistance in human bladder cancer cells. Mol Med Rep.

[102] Almeida TC, Guerra CCC, De Assis BLG, de Soares RD, Garcia CCM, Lima AA, da Silva GN (2019). Antiproliferative and toxicogenomic effects of resveratrol in bladder cancer cells with different TP53 status. Environ Mol Mutag.

[103] Zhou C, Ding J, Wu Y (2014). Resveratrol induces apoptosis of bladder cancer cells via miR-21 regulation of the Akt/Bcl-2 signaling pathway. Mol Med Rep.

[104] Hong L, Han Y, Zhang Y, Zhang H, Zhao Q, Wu K, Fan D (2013). MicroRNA-21: a therapeutic target for reversing drug resistance in cancer. Exp Opin Ther Targets.

[105] Li L, Chen D, Zhu J, Ouyang J, Song B, Shenglong Z, KaiShun L, Wei L, Yun L (2019). Inhibition of resveratrol on bladder cancer and its molecular mechanism. Acta Medica Mediterranea.

[106] Jing X, Cheng W, Wang S, Li P, He L (2016). Resveratrol induces cell cycle arrest in human gastric cancer MGC803 cells via the PTEN-regulated PI3K/Akt signaling pathway. Oncol Rep.

[107] Zheng M, Chen R, Zhong H, Lin Q, Wang X, Zhao Z, Xie L (2012). Side-effects of resveratrol in HepG2 cells: reduced pten and increased bcl-xl mRNA expression. Mol Med Rep.

[108] Dhar S, Kumar A, Rimando AM, Zhang X, Levenson AS (2015). Resveratrol and pterostilbene epigenetically restore PTEN expression by targeting oncomiRs of the miR-17 family in prostate cancer. Oncotarget.

[109] Kumar A, Rimando AM, Levenson AS (2017). Resveratrol and pterostilbene as a microRNA-mediated chemopreventive and therapeutic strategy in prostate cancer. Ann N Y Acad Sci.

[110] Davidov-Pardo G, McClements DJ (2014). Resveratrol encapsulation: designing delivery systems to overcome solubility, stability and bioavailability issues. Trends Food Sci Technol.

[111] Venditti I, Iucci G, Fratoddi I, Cipolletti M, Montalesi E, Marino M, Secchi V, Battocchio C (2020). Direct conjugation of resveratrol on hydrophilic gold nanoparticles: structural and cytotoxic studies for biomedical applications. Nanomaterials.

[112] Chamsai B, Samprasit W, Opanasopit P, Benjasirimongkol P, Sriamornsak P (2020). Types of solid lipids on physical stability of resveratrol-loaded nanostructured lipid carriers. Key Eng Mater..

[113] Sharifi-Rad J, Rayess Y, Rizk A, Sadaka C, Zgheib R, Zam W, Sestito S, Rapposelli S, Neffe-Skocińska K, Zielińska D (2020). Turmeric and its major compound curcumin on health: bioactive effects and safety profiles for food, pharmaceutical, biotechnological and medicinal applications. Front Pharmacol.

[114] Mirzaei H, Masoudifar A, Sahebkar A, Zare N, Sadri Nahand J, Rashidi B, Mehrabian E, Mohammadi M, Mirzaei HR, Jaafari MR (2018). MicroRNA: a novel target of curcumin in cancer therapy. J Cell Physiol.

[115] Sun M, Estrov Z, Ji Y, Coombes KR, Harris DH, Kurzrock R (2008). Curcumin (diferuloylmethane) alters the expression profiles of microRNAs in human pancreatic cancer cells. Mol Cancer Ther.

[116] Hatab HM, Hamid FFA, Soliman AF, Al-Shafie TA, Ismail YM, El-Houseini ME (2019). A combined treatment of curcumin, piperine, and taurine alters the circulating levels of IL-10 and miR-21 in hepatocellular carcinoma patients: a pilot study. J Gastrointest Oncol.

[117] Lelli D, Pedone C, Sahebkar A (2017). Curcumin and treatment of melanoma: the potential role of microRNAs. Biomed Pharmacother.

[118] Xu R, Li H, Wu S, Qu J, Yuan H, Zhou Y, Lu Q (2019). MicroRNA-1246 regulates the radio-sensitizing effect of curcumin in bladder cancer cells via activating P53. Int Urol Nephrol.

[119] Saini S, Arora S, Majid S, Shahryari V, Chen Y, Deng G, Yamamura S, Ueno K, Dahiya R (2011). Curcumin modulates MicroRNA-203-mediated regulation of the Src-Akt axis in bladder cancer. Cancer Prev Res.

[120] Wang K, Tan S-L, Lu Q, Xu R, Cao J, Wu S-Q, Wang Y-H, Zhao X-K, Zhong Z-H (2018). Curcumin suppresses microRNA-7641-mediated regulation of p16 expression in bladder cancer. Am J Chin Med.

[121] Network CGAR (2014). Comprehensive molecular characterization of urothelial bladder carcinoma. Nature.

[122] Liu ST, Hui G, Mathis C, Chamie K, Pantuck AJ, Drakaki A (2018). The current status and future role of the phosphoinositide 3 kinase/AKT signaling pathway in urothelial cancer: an old pathway in the new immunotherapy era. Clin Genitourinary Cancer.

[123] Winters BR, Vakar-Lopez F, Brown L, Montgomery B, Seiler R, Black PC, Boormans JL, Dall M, Davincioni E, Douglas J (2018). Mechanistic target of rapamycin (MTOR) protein expression in the tumor and its microenvironment correlates with more aggressive pathology at cystectomy. Urol Oncol Semin Orig Investig..

[124] Liu J, Zeng Q, Cao P, Xie D, Yang F, He L, Dai Y, Li J, Liu X, Zeng H (2018). SPAG5 promotes proliferation and suppresses apoptosis in bladder urothelial carcinoma by upregulating Wnt3 via activating the AKT/mTOR pathway and predicts poorer survival. Oncogene.

[125] Tian B, Zhao Y, Liang T, Ye X, Li Z, Yan D, Fu Q, Li Y (2017). Curcumin inhibits urothelial tumor development by suppressing IGF2 and IGF2-mediated PI3K/AKT/mTOR signaling pathway. J Drug Target.

[126] Wang J, Wang Z, Wang H, Zhao J, Zhang Z (2011). Curcumin induces apoptosis in ej bladder cancer cells via modulating c-myc and pi3k/akt signaling pathway. World J Oncol.

[127] Chang M, Wu M, Li H (2020). Antitumor effects of curcumin and glycyrrhetinic acid-modified curcumin-loaded cationic liposome by intratumoral administration. Evid Based Complement Alternative Med..

[128] El-Shafey AA, Hegab MH, Seliem MM, Barakat AM, Mostafa NE, Abdel-Maksoud HA, Abdelhameed RM (2020). Curcumin@ metal organic frameworks nano-composite for treatment of chronic toxoplasmosis. J Mater Sci Mater Med.

[129] Weaver BA (2014). How Taxol/paclitaxel kills cancer cells. Mol Biol Cell.

[130] Wei Y, Pu X, Zhao L (2017). Preclinical studies for the combination of paclitaxel and curcumin in cancer therapy. Oncol Rep.

[131] Zhao Y-f, Han M-l, Xiong Y-j, Wang L, Fei Y, Shen X, Zhu Y, Liang Z-q (2018). A miRNA-200c/cathepsin L feedback loop determines paclitaxel resistance in human lung cancer A549 cells in vitro through regulating epithelial–mesenchymal transition. Acta Pharmacol Sinica.

[132] Lu C, Xie Z, Peng Q (2017). MiRNA-107 enhances chemosensitivity to paclitaxel by targeting antiapoptotic factor Bcl-w in non small cell lung cancer. Am J Cancer Res.

[133] Wang S-Q, Wang C, Chang L-M, Zhou K-R, Wang J-W, Ke Y, Yang D-X, Shi H-G, Wang R, Shi X-L (2016). Geridonin and paclitaxel act synergistically to inhibit the proliferation of gastric cancer cells through ROS-mediated regulation of the PTEN/PI3K/Akt pathway. Oncotarget.

[134] Papadopoulos EI, Scorilas A (2015). Cisplatin and paclitaxel alter the expression pattern of miR-143/145 and miR-183/96/182 clusters in T24 bladder cancer cells. Clin Transl Sci.

[135] Kunnumakkara AB, Harsha C, Banik K, Vikkurthi R, Sailo BL, Bordoloi D, Gupta SC, Aggarwal BB (2019). Is curcumin bioavailability a problem in humans: lessons from clinical trials. Exp Opin Drug Metab Toxicol.

[136] Hsieh C (2001). Phase I clinical trial of curcumin, a chemopreventive agent, in patients with high-risk or pre-malignant lesions. Anticancer Res.

[137] Garcea G, Berry DP, Jones DJ, Singh R, Dennison AR, Farmer PB, Sharma RA, Steward WP, Gescher AJ (2005). Consumption of the putative chemopreventive agent curcumin by cancer patients: assessment of curcumin levels in the colorectum and their pharmacodynamic consequences. Cancer Epidemiol Prev Biomark.

[138] Sharma RA, Euden SA, Platton SL, Cooke DN, Shafayat A, Hewitt HR, Marczylo TH, Morgan B, Hemingway D, Plummer SM (2004). Phase I clinical trial of oral curcumin: biomarkers of systemic activity and compliance. Clin Cancer Res.

[139] Durgaprasad S, Pai CG, Alvres JF (2005). A pilot study of the antioxidant effect of curcumin in tropical pancreatitis. Indian J Med Res.

[140] Roth BJ (1995). Preliminary experience with paclitaxel in advanced bladder cancer. Semin Oncol.

[141] Albers P, Park S-I, Niegisch G, Fechner G, Steiner U, Lehmann J, Heimbach D, Heidenreich A, Fimmers R, Siener R (2011). Randomized phase III trial of 2nd line gemcitabine and paclitaxel chemotherapy in patients with advanced bladder cancer: short-term versus prolonged treatment [German Association of Urological Oncology (AUO) trial AB 20/99]. Ann Oncol.

[142] Paz-Ares L, Solsona E, Esteban E, Saez A, Gonzalez-Larriba J, Anton A, Hevia M, de la Rosa F, Guillem V, Bellmunt J (2010). Randomized phase III trial comparing adjuvant paclitaxel/gemcitabine/cisplatin (PGC) to observation in patients with resected invasive bladder cancer: results of the Spanish Oncology Genitourinary Group (SOGUG) 99/01 study. J Clin Oncol.

[143] Sridhar SS, Blais N, Tran B, Reaume MN, North SA, Stockler MR, Chi KN, Fleshner NE, Liu G, Robinson JW (2020). Efficacy and Safety of nab-Paclitaxel vs paclitaxel on survival in patients with Platinum-refractory metastatic urothelial cancer: the Canadian Cancer Trials Group BL. 12 Randomized Clinical Trial. JAMA Oncol.

[144] Singh AP, Singh R, Verma SS, Rai V, Kaschula CH, Maiti P, Gupta SC (2019). Health benefits of resveratrol: evidence from clinical studies. Med Res Rev.

[145] Howells LM, Berry DP, Elliott PJ, Jacobson EW, Hoffmann E, Hegarty B, Brown K, Steward W, Gescher AJ (2011). Phase I randomized, double-blind pilot study of micronized resveratrol (SRT501) in patients with hepatic metastases—safety, pharmacokinetics, and pharmacodynamics. Cancer Prev Res.

[146] Patel KR, Brown VA, Jones DJ, Britton RG, Hemingway D, Miller AS, West KP, Booth TD, Perloff M, Crowell JA (2010). Clinical pharmacology of resveratrol and its metabolites in colorectal cancer patients. Cancer Res.

[147] Kjær TN, Ornstrup MJ, Poulsen MM, Jørgensen JOL, Hougaard DM, Cohen AS, Neghabat S, Richelsen B, Pedersen SB (2015). Resveratrol reduces the levels of circulating androgen precursors but has no effect on, testosterone, dihydrotestosterone, PSA levels or prostate volume. A 4-month randomised trial in middle-aged men. Prostate.

[148] Paller CJ, Rudek MA, Zhou XC, Wagner WD, Hudson TS, Anders N, Hammers HJ, Dowling D, King S, Antonarakis ES (2015). A phase I study of muscadine grape skin extract in men with biochemically recurrent prostate cancer: safety, tolerability, and dose determination. Prostate.

[149] Popat R, Plesner T, Davies F, Cook G, Cook M, Elliott P, Jacobson E, Gumbleton T, Oakervee H, Cavenagh J (2012). A phase 2 study of SRT501 (resveratrol) with bortezomib for patients with relapsed and or refractory multiple myeloma. Br J Haematol.

[150] Dhillon N, Aggarwal BB, Newman RA, Wolff RA, Kunnumakkara AB, Abbruzzese JL, Ng CS, Badmaev V, Kurzrock R (2008). Phase II trial of curcumin in patients with advanced pancreatic cancer. Clin Cancer Res.

